# Determination of Coverage Interval of Antioxidant Vitamins (Vitamin C & Vitamin E) in Plasma and Serum of Bengali Population

**DOI:** 10.1155/2013/590791

**Published:** 2013-11-14

**Authors:** Sanghamitra Chakraborty, Indranil Chakraborty

**Affiliations:** ^1^Department of Biochemistry, Medical College, 88th College Street, Kolkata 700073, India; ^2^Department of Biochemistry, Malda Medical College, Englishbazar, Dist-Malda, West Bengal 732101, India

## Abstract

Reference interval of all haematological and biological analytes should be measured for every population because of the huge diversity in genetic make-up, dietary habits. The coverage interval of antioxidant vitamins (vitamin C and **α**-tocopherol) in the plasma and serum of reference Bengali population was determined and compared with the reference intervals of antioxidant vitamins in the established literature. Adult healthy volunteers from 18 to 68 years of age underwent extensive clinical and investigational procedure and were included in the study. Vitamin C and **α**-tocopherol were estimated using simple Spectrophotometric method. Of the 71 healthy Bengali volunteers participated, 31 were males and 40 were females. The mean concentration of plasma vitamin C was found to be 0.65 mg/dL. The mean **α**-tocopherol was found 6.35 mg/L (14.74 **μ**mol/L) in the study population higher than the normal threshold value for **α**-tocopherol but lower than other populations. The study data enabled us to determine the gender nonspecific coverage interval of antioxidant vitamins, and the intervals were lower than the established reference interval in other populations.

## 1. Introduction

In recent years, with the increased dependence on laboratory tests, the sound understanding of reference intervals is vital as diagnosis of disease frequently depends on results of laboratory analytes measured from blood, urine, cerebrospinal fluid, and so forth. Reference interval denotes normative values related to laboratory parameters used by the clinical laboratories for clinical diagnosis [1]. The concept of reference values was introduced in 1969 by Grasbeck and Saris [2]. However there is often an overlap between normal and abnormal values as most disease and biological analytes change in a continuing fashion. So, the concept of normal value is now considered to be ambiguous [3]. Moreover, biological data from a reference sample is skewed; the use of the normal terminology may be misleading by implying that the distribution is bellshaped symmetrical Gaussian distribution [4]. According to the international recommendations, every country or clinical laboratory must establish reference interval for healthy individuals belonging to a group of homogeneous population (CLIA′88 regulation) [5]. Population based reference interval comprises central 95% of healthy individual located between 0.025 and 0.975 fractiles as defined by ISO 15189 and IFCC (International Federation of Clinical Chemistry) [6]. According to IFCC recommendation, calculation of reference interval requires measurement of at least 120 samples by nonparametric methods [7]. However it is always not possible to obtain 120 healthy reference individuals for all analytes in a cost effective manner based on the stringent inclusion and exclusion criteria. The coverage interval (also called the reference interval in clinical chemistry) refers to population based reference values obtained from a well-defined (healthy) group of reference individuals. This is an interval with two confidence limits that cover the individual values in the population in some probabilistic sense. The coverage probability of a confidence interval is the proportion of time that the interval contains the true values of interest [8]. Recently, IUPAC (International Union of Pure and Applied Chemistry) have advocated the use of *Coverage interval* where it can be stated with a given degree of confidence that the interval contains a given fraction of the population of reference values [9]. The coverage interval may be used not only to provide a meaningful interval when few reference values are available, but also, with many reference values, to provide useful information on the precision of the estimated interval.

In both health and disease, increasing interest is being directed to the significance of optimal vitamin and antioxidant status [10]. Antioxidant status evidently varies considerably among populations. Impaired antioxidant status has been identified in several disorders, such as cholestatic liver disease, exocrine pancreatic insufficiency, nutritional deprivation due to protein calorie malnutrition, and acquired immunodeficiency syndrome (AIDS) Intervention [11]. Low plasma Vitamin C concentrations are also reported in patients with diabetes, infections, and in smokers, but the relative contribution of diet and stresses to these situations are uncertain. Epidemiologic studies indicate that diets with high vitamin C content have been associated with lower cancer risk, especially for cancers of the oral cavity, esophagus, stomach, colon, and lung [12]. Data regarding reference interval of these antioxidant vitamins like other haematological and biochemical parameters may not be applicable to the Indian population because of enormous racial and ethnic diversity [13]. The objective of the present work is to determine the coverage interval of antioxidant vitamins (vitamin C and vitamin E) in the serum and plasma of reference Bengali individuals of West Bengal.

## 2. Materials and Methods

Seventy one healthy Bengalis, thirty one males (M = 31) and forty females (F = 40), in the age group between 18 to 68 years (mean 28.49 years) were enrolled. The study subjects had ordinary eating habits with none on a special diet or vitamin supplements. The staple diet consists of rice, vegetables, egg, and fish. Fruits were not very prominent but there was ample consumption of vegetables like pumpkin and carrot. The dietary pattern was enquired by a less accurate model questionnaire. Subjects with hypertension, hypercholesterolemia, diabetes, obesity, genetically determined risks, habits of smoking, alcohol consumption, any history of drug intake for treatment of disease or suffering like antiepileptic, ATD (anti-tubercular drugs), Chemotherapy, OCP (oral contraceptive pills), any variation in physiological status like pregnancy, stress, and excessive exercise were excluded from the study. Following overnight fasting, peripheral venous samples were taken into evacuated tubes, centrifuged, and plasma and sera were separated. The analysis of the antioxidant vitamins were done in accordance with the standards of the Ethical Committee of Medical College, Kolkata. Estimation of *α*-tocopherol in serum has been done by Baker and Frank method (1968) [14]. Plasma vitamin C was estimated by Roe & Kuether method [15]. In spite of the fact that well-established spectrophotometric methodologies were used in our research work, the methodology was compared with current reference method, that is, HPLC (High performance liquid chromatography) and evaluated in-house for the assessment of performance. Both of the spectrophotometric methodologies were assesed individualy by the estimation of Precision, recovery analysis (using spiked sample), and proportional error in house. It is evident from Table 1 that the performance parameter of both spectrophotometric methods used in our study is within the limits of total allowable error.

Moreover, the traceability of the methodology was calculated by comparing with an established HPLC from an accredited laboratory. For this purpose, randomised samples from the study population were measured in parallel and compared with an established HPLC method from an accredited laboratory, for *α* tocopherol the calculated Bias was −5.29%. The HPLC method for *α*-tocopherol was having the following performance criteria as linearity: 0.2–50 mg/L, recovery rate: 92–98%, lower detection limit: 0.30 mg/L, lower determination limit: 0.40 mg/L, interassay coefficient of variance: 3.23% and reference interval = 5–18 mg/L. Similarly, for the vitamin C the bias and exactness were −1.79% and 98.21%, respectively, after comparing the value of samples from the study population with an established HPLC method from an accredited laboratory.

## 3. Results and Discussion

The values of the antioxidant vitamins were analysed using IBM SPSS 16 statistical software. The Kolmogorov-Smirnov test was used to check for normal distribution. Both “parametric” and “nonparametric” coverage intervals were calculated. The mean age and weight of the study population 28.49 ± 9.49 years and 62.9 ± 11.58 Kilograms, respectively.

It is evident from Table 2 that the mean concentration of plasma vitamin C was found to be 0.65 mg/dL and 95% confidence interval was 0.65 ± 0.051 mg/dL. The reference interval for plasma ascorbic acid as per 2002 CLSI C28-A2 guide line is 0.4–1.5 mg/dL. According to Table 2, the “parametric coverage interval” and “nonparametric coverage interval” was found to be 0.32–1.17 mg/dL and 0.35–1.2 mg/dL, respectively. The coverage interval provides here a useful image of the reference interval and distribution, but the limits of coverage interval is lower than established reference limits. Moreover, from technical considerations of the Roe and Kuther [15] methodology used in this study, the bias and proportional error was calculated to be −1.79% and 9.45%, respectively. This was within the acceptable limits documented by Ricós et al. [16]. According to them, the most recent and extensive listing of biological goals for plasma Vitamin C should be 20% within subject biological variation, 21% between subject biological variation, 10% imprecision, 7.3% bias, and 23.8% allowable total error. Thus, the methodology was found to be acceptable and quite robust.

From Table 3, the mean concentration of *α*-tocopherol was found 6.35 mg/L (14.74 *μ*mol/L) in the study population. The cut-off value for serum *α*-tocopherol ≥ 11.6 *μ*mol/L, subjects with serum concentration less than this are considered deficient. Comparison with studies done on Spanish, Italian, American, Japanese, Hispanic, Swiss, and Kuwaiti population suggested that the mean concentration of *α*-tocopherol of these study subjects was much lower. We observed the mean concentration of *α*-tocopherol to be 28.2 *μ*mol/L, 26.2 *μ*mol/L, 25.7 *μ*mol/L, 24.4 *μ*mol/L, 29.2 *μ*mol/L, 29.3 *μ*mol/L, and 20.0 *μ*mol/L in Spanish [17], Italian [18], American [19], Japanese [20], Hispanic [21], Swiss [22], and Kuwaiti [23] population, respectively.

Moreover, *α*-tocopherol showed greatest association with the lipid, an observation confirmed by the Swiss study of Winklhofer-Roob et al. [24]. The concentration of *α*-tocopherol is found to be more concentrated in the hydrophobic plasma membrane and phospholipid coats of lipoproteins. *α*-tocopherol has an well-established role against LDL oxidation. As the plasma concentration of *α*-tocopherol varies with cholesterol profile it is rationalised to estimate lipid standardised Vitamin E and express Vitamin E as *μ*mol/L of tocopherol/mmol/L of cholesterol [25]. Using regression analysis, 1.11 *μ*mol tocopherol/mmol total lipids were calculated as the threshold of deficiency equivalent to 0.8 mg tocopherol/g total lipid established by Horwitt et al.

From Table 3, the parametric and non-parametric coverage interval of *α* tocopherol was found to be 3–11.9 mg/L and 3.3–13.3 mg/L, respectively. This is not in agreement with the established reference intervals found in the literature, that is, 5–18 mg/L in adults. The oxidative stress varies with age. In this study, the age group was 18–68 years, so naturally the concentration of *α*-tocopherol varied in accordance with age and corresponding oxidative stress. The literature survey revealed that all the studies done on Swiss, Kuwaitis, and Spanish population employed HPLC, the method of choice for quantification. According to the most recent and extensive listing of biological goals provided by Ricós et al., 13.8% within subject biological variation, 15% between subject biological variation, 6.9% imprecision, 5.1% bias, and 16.5% allowable total error are permissible for serum *α* tocopherol [16]. The photometric method used in our study had a negative bias of 5.29% which is slightly greater than recommended 5.1%. So, the method used in our study detected some-what lesser concentration than actual value. But the proportional error was 8%, that is, lesser than the total allowable error. Thus, the method was acceptable. Moreover, the assessment of performance parameters used in this study reflects the reliability of the study.

## 4. Conclusion

This study indicates that the coverage interval obtained from the Bengali population can be used to define antioxidant reference thresholds. In spite of the limitation imposed by cost and methodology like HPLC, the study has demonstrated that the plasma concentration of vitamin C and *α* tocopherol in most instances specific to Bengali population. To establish more credible “coverage interval” for these antioxidant vitamins as a replica of reference interval, a similar study with improved method of estimation like HPLC is warranted. It was established that western normative laboratory values of vitamin C and Vitamin E are not applicable to Bengali population. This finding provides an insight that not only methods of estimation, but also on different ethnicity, racial, genetic make-up, sociodemographic pattern and dietary habits.

## Figures and Tables

**Table 1:** Showing the inhouse performance parameter of the spectrophotometric methods of Vitamin C and *α*-tocopherol.

Name of the Analyte	Performance Parameter	Percentage (%)
Vitamin C	Precision	6
	Recovery Analysis from Spike sample	90.55
	Proportional Error	9.45 (<23.8%, Total allowable error) [16]
	Sensitivity (*R*_2_)	0.9965

*α*-tocopherol	Precision	6.65
	Recovery Analysis from Spike sample	92
	Proportional Error	8 (<16.5%, Total allowable error) [16]

**Table 2:** Distribution of reference values of vitamin C.

Number of observations (*n*)	71
Distribution of reference values (mg/dL)	
Mean	0.65
Median	0.6
95% CI (confidence interval)	0.65 ± 0.05
Test for normal distribution	
Kolmogorov Smirnov^a^ test. *P*	0.006^#^
Non parametric Coverage interval	0.35–1.2 mg/dL
Kolmogorov Smirnov test after logarithmic transformation. *P*	0.2*
Parametric coverage interval	0.32–1.17 mg/dL

^#^The distribution is not in normal distribution as *P* < 0.05.

*This is a lower bound true significance *P* > 0.05.

^a^Refers to in statistics, the Lilliefors test.

**Table 3:** Distribution of Reference Values of *α*-tocopherol.

Number of observations (*n*)	71
Distribution of reference values (mg/L)	
Mean	6.35
Median	5.99
95% CI (confidence interval)	6.35 ± 0.54
Test for normal distribution	
Kolmogorov Smirnov^a^ test. *P*	0.037^#^
Non parametric Coverage interval	3.3–13.3 mg/L
Kolmogorov Smirnov test after logarithmic transformation. *P*	0.200*
Parametric coverage interval	3–11.9 mg/L

^#^The distribution is not in normal distribution as *P* < 0.05.

*This is a lower bound true significance *P* > 0.05.

^a^Refers to in statistics, the Lilliefors test.
